# Identification and Characterization of a Novel HIV-1 Circulating Recombinant Form (CRF59_01B) Identified among Men-Who-Have-Sex-with-Men in China

**DOI:** 10.1371/journal.pone.0099693

**Published:** 2014-06-30

**Authors:** Weiqing Zhang, Xiaoxu Han, Minghui An, Bin Zhao, Qinghai Hu, Zhenxing Chu, Jiancheng Xu, Weiping Cai, Xi Chen, Jihua Fu, Zhe Wang, Jianjun Wu, Lin Lu, Minghua Zhuang, Hao Wu, Hongjing Yan, Christina Liao, Yutaka Takebe, Hong Shang

**Affiliations:** 1 Key Laboratory of AIDS Immunology of National Health and Family Planning Commission, Department of Laboratory Medicine, The First Hospital of China Medical University, Shenyang, China; 2 Collaborative Innovation Center for Diagnosis and Treatment of Infectious Diseases, Hangzhou, China; 3 AIDS Research Center, National Institute of Infectious Diseases, Tokyo, Japan; 4 Infectious Disease Department, Guangzhou No. 8 Renmin Hospital, Guangzhou, China; 5 AIDS/STIs Prevention and Control Department, Hunan Provincial Center for Disease Control and Prevention, Changsha, China; 6 Sexually transmitted Disease and AIDS Department, Shandong Provincial Center for Disease Control and Prevention, Jinan, China; 7 Henan Provincial Center for Disease Control and Prevention, Zhengzhou, China; 8 Sexually transmitted Disease and AIDS Department, Anhui Provincial Center for Disease Control and Prevention, Hefei, China; 9 Yunnan Provincial Center for Disease Control and Prevention, Kunming, China; 10 Sexually transmitted Disease and AIDS Department, Shanghai Municipal Center for Disease Control and Prevention, Shanghai, China; 11 Infectious Diseases Department, Beijing Youan Hospital, Capital Medical University, Beijing, China; 12 Sexually Transmitted Disease and AIDS Prevention and Control Department, Jiangsu Provincial Center for Disease Control and Prevention, Nanjing, China; Shanghai Medical College, Fudan University, China

## Abstract

The HIV-1 epidemic among men-who-have-sex-with-men (MSM) continues to expand in China. A large-scale national survey we conducted on HIV-1 strains among MSM in 11 provinces in China from 2008 to 2013 (*n* = 920) identified a novel transmission cluster consisting of six strains (0.7%) that belonged to a new circulating recombinant form (designated CRF59_01B). CRF59_01B contains two subtype B segments of U.S.-European origin (in the *pol* and *vpu-env* regions) in a CRF01_AE backbone. CRF59_01B is the second CRF (after CRF55_01B) circulating primarily among MSM in China. CRF59_01B occurs at a low frequency (less than 1%), but it was detected in four different provinces/regions in China: Liaoning (northeast China) (n = 3); Hunan (central China) (n = 1); Guangdong (south China) (n = 1); Yunnan (southwest China) (n = 1). One additional recombinant strain was detected in a heterosexual individual in Liaoning province but is not the focus of this paper. Bayesian molecular clock analyses indicate that CRF59_01B emerged as a result of recombination between CRF01_AE and subtype B around the year 2001. The emergence of multiple forms of recombinants and CRFs reflects the ever-increasing contribution of homosexual transmission in China's HIV epidemic and indicates an active HIV transmission network among MSM in China.

## Introduction

A high level of genetic diversity is the hallmark of human immunodeficiency virus type 1 (HIV-1). HIV-1 is classified into four groups: M, O, N and P [1], [2]. HIV-1 group M strains are responsible for the vast majority of HIV infections worldwide and consist of 11 subtypes and sub-subtypes, 58 circulating recombinant forms (CRFs) (www.hiv.lanl.gov), and various types of unique recombinant forms (URFs). A variety of CRFs and URFs continue to be detected worldwide. In particular, new CRF strains with serial numbers above 51 were all reported from Asia: CRF51_01B from Singapore [3]; CRF52_01B from Thailand and Malaysia [4]; CRF53_01B [5] and CRF54_01B [6] from Malaysia; and CRF55_01B [7] from China. All CRFs reported from Asia are recombinants of CRF01_AE and subtype B, except CRF07_BC [8] and CRF08_BC [9]. Widespread co-circulation and dual infection of CRF01_AE and subtype B in various regions in Asia have led to the emergence of a large number of CRFs comprising subtype B and CRF01_AE. Of note among them, CRF51_01B and CRF55_01B are CRFs that were identified mainly among men-who-have-sex-with-men (MSM).

The HIV-1 epidemic continues to expand rapidly among MSM in China [10]–[12]. In 2011, MSM accounted for 29.4% of all newly diagnosed HIV cases [10]. MSM became one of the nation's most targeted populations for HIV prevention and care. Initially, the MSM population in China was predominantly infected with subtype B [13], [14], the typical U.S.-European strains that are prevalent in western countries. However, in recent years, a dramatic shift in genotype distribution from subtype B to CRF01_AE and other virus lineages has been observed in China [15]. Furthermore, studies [16], [17] have identified a number of distinct phylogenetic clusters uniquely associated with the epidemic among MSM in China: CRF01_AE clusters 1 and 2, and CRF07_BC cluster 3. These three lineages of HIV-1 strains account for approximately 80% of HIV-1 infections among MSM in China [17]. In addition, various new recombinant strains mostly comprising CRF01_AE and subtype B have been detected [18], [19].

In the present study, we discuss a new circulating recombinant form that we identified (CRF59_01B) and that is uniquely associated with transmission among MSM in China. Additionally, we investigate its nationwide occurrence and the evolutionary history of it emergence.

## Materials and Methods

### Study Subjects, HIV-1 RNA Isolation and Screening of HIV-1 Genotypes

This study was performed as part of a nationwide molecular epidemiological survey of Chinese MSM. A total of 920 plasma samples were collected from HIV-1-seropositive MSM in 11 provinces/municipalities across China from 2008 to 2013: Jilin province (n = 8); Liaoning province (n = 263); Beijing (n = 163); Shandong province (n = 42); Jiangsu province (n = 49); Shanghai (n = 26); Anhui province (n = 136); Henan province (n = 58); Hunan province (n = 68); Guangdong province (n = 40); and Yunnan province (n = 67). This study was approved by the Institutional Review Board of the First Affiliated Hospital of China Medical University. Written informed consent was obtained from all participants before sample collection. HIV-1 RNA was extracted from participants' plasma using QIAamp Viral Mini Kits (Qiagen, Germany) and was used to amplify and determine the nucleotide sequences of the 1.1-kb protease-reverse transcriptase (pro-RT) region in the *pol* gene (HXB2: 2253–3318). HIV-1 genotypes were determined based on the neighbor-joining analysis of the Kimura 2-parameter distance matrix and a transition-to-transversion ratio of 2.0, using MEGA software Version 5.0

### Near Full-Length HIV- 1 Nucleotide Sequencing

Near-full-length genome (NFLG) sequences of the strains of interest were determined using the single-gene amplification (SGA) method [20] to prevent any artificial recombination that might have occurred with a nested polymerase chain reaction (PCR). Briefly, plasma HIV-1 RNA was reverse-transcribed into single-strand cDNA using Superscript III First-Strand Synthesis System (Invitrogen, USA) with 5′-half -reverse primer 07Rev8 (5′-CCTARTGGGATGTGTACTTCTGAACTT-3′; HXB2: 5193–5219 nt) and 3′–half-reverse primer 1.R3.B3R (5′-ACTACTTGAAGCACTCAAGGCAAGCTTTATTG-3′; HXB2: 9611–9642 nt) as described previously [21]. The 5′- and 3′- halves of the HIV-1 viral genome were independently amplified from cDNA by using two rounds of nested PCR with specific primers [21]. Both PCR reactions were performed in a final volume of 20 ul containing 15.3 ul RNase-free Water, 2 ul 10× High-Fidelity Platinum PCR buffer, 0.8 ul MgSO_4_ (50 mM), 0.4 ul dNTP (10 mM), 0.2 ul of each primer (20 pmol/ul), 0.1 ul Platinum Taq High-Fidelity polymerase (Invitrogen, USA), and 1 ul template. The first and second rounds of PCR were both performed under the following conditions: 94°C for 2 minutes, 35 cycles at 94°C for 10 seconds, 60°C for 30 seconds, 68°C for 4.5 minutes, final extension of 10 minutes at 68°C. The second-round PCR products were electrophoresed on 0.7% TAE agarose gel to check for positive amplification. Then, the positive amplification products were purified and directly sequenced using internal walking primers with an ABI 3730XL Sanger-based genetic analyzer. All sequences were analyzed, edited and assembled by overlapping the sequences of the two half-genome fragments with Sequencer 4.10.1 and Bioedit version 5.0.

### Recombination breakpoint analyses

The NFLG sequences were first analyzed using the Recombination Identification Program (RIP) and the jumping profile Hidden Markov Model (jpHMM) on the Los Alamos HIV Sequence Database (www.hiv.lanl.gov) to define the recombinant structures. Subsequently, all NFLG sequences were aligned with HIV-1 subtypes/CRFs reference sequences using HIVAlign (http://www.hiv.lanl.gov/content/sequence/VIRALIGN/viralign.html) and then manually edited with Bioedit 5.0. A phylogenetic tree of the NFLG was constructed by applying the neighbour-joining method based on Kimura's two-parameter distance matrix with 1000 bootstrap replicates using MEGA 5.0. Subtype B (83FR.HXB2), CRF01_AE (90TH.CM240) and subtype C (95IN21068) were used in the bootscanning analysis with SimPlot version 3.5.1. Insertion segments were used to build sub-regions phylogenetic tree via the neighbour-joining method with bootstrapping to confirm the origin of the different segments. The Recombinant HIV-1 Drawing Tool (http://www.hiv.lanl.gov/content/sequence/DRAWCRF/recom_mapper.html) was used to elucidate the structure of the new HIV-1 recombinant forms (CRF01_AE/B).

### Estimate of Appearance of Most Recent Common Ancestor of CRF59_01B

The rate of the evolution of different segments of CRF59_01B were estimated from a set of subtype B and CRF01_AE references with known sampling dates using BEAST v.1.6.0. Dates were estimated using Bayesian Markov Chain Monte Carlo (MCMC) inference under both the general time-reversal (GTR) and Hasegawa-Kishino-Yano (HKY) nucleotide substitution models. The MCMC analysis was computed for 20 million states sampled at every 1000 states, and the MCMC results were evaluated using the Tracer 1.5 program. All parameters were estimated from an effective sampling size (ESS) >200. The maximum clade credibility (MCC) trees were viewed and edited using FigTree v1.3.1.

## Results

### Identification of Novel Circulating Recombinant Form (CRF59_01B) among MSM in China

**Figure 1:**
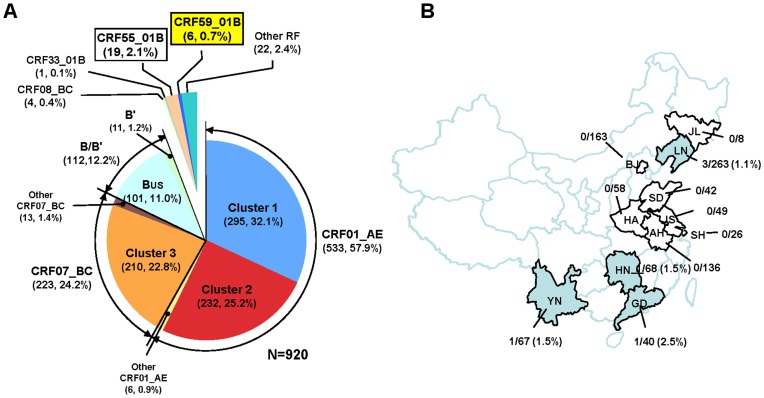
HIV-1 genotype distribution among MSM in China and map of China depicting the occurrence of CRF59_01B. (A) The overall genotype distribution of HIV-1 strains among MSM newly diagnosed during 2008–2013 in 11 provinces in China (n = 920). (B) Map of China. Study sites (11 provinces) and the proportion of CRF59_01B among HIV-1 strains in their respective provinces.

The primary screening of HIV-1 genotypes based on 1.1-kb *pol* (pro-RT) region sequences revealed that the genotype distribution of HIV-1 strains circulating among MSM in 11 provinces in China (n = 920) were as depicted in Figure 1A: CRF01_AE (533, 57.9%); CRF07_BC (223, 24.2%); B (101, 11.0%); B′ (Thai variant of subtype B) (11, 1.2%); CRF08_BC (4, 0.4%); CRF33_01B (1, 0.1%); other recombinants (47, 5.1%). Furthermore, CRF01_AE strains were classified into two distinct clusters [16], [17]: cluster 1 (295, 32.1%) and cluster 2 (232, 25.2%). Similarly, most CRF07_BC strains formed a unique phylogenetic cluster that is designated cluster 3 (210, 22.8%) [17]. Additionally, 19 strains (2.1%) belong to the recently-identified CRF55_01B strain [7].

Within a group of other recombinant strains (n = 28), we found that six strains (0.7%) formed a distinct phylogenetic cluster that is different from any other known HIV-1 genotype: 3 from Liaoning province (northeast), and 1 each from Guangdong, Yunnan and Hunan provinces (Table 1, Figure 1B). Furthermore, we also identified one additional strain (11CN.LNSY300876) that belonged to this cluster from a heterosexual male in Liaoning province (Table 1). These seven study subjects share no obvious epidemiologic link. Recombination breakpoint analysis revealed that these seven strains contained a small subtype B segment in a CRF01_AE backbone in the 1.1-kb *pol* segment (data not shown).

**Table 1:** Summary of demographic and genotype information of study subjects infected with CRF59_01B in China.

Study subject	Geographic origin Province (City)	Sampling year	Age	Sex	Risk factor	Genome sequence	Accession number	Remarks
09CN.LNSY300423	Liaoning (Shenyang)	2009	51	M	MSM	NFLG	JX960635	Cite [16]
10CN.LNSY300533	Liaoning (Shenyang)	2010	31	M	Bisexual	NFLG	KC462191	Cite [30]
11CN.LNSY300392	Liaoning (Shenyang)	2011	51	M	MSM	NFLG	KC462190	Cite [30]
11CN.GDMM152	Guangdong (Maoming)	2011	40	M	MSM	NFLG	KJ484433	This study
12CN.YNKM200199	Yunnan (Kunming)	2012	43	M	MSM	NFLG	KJ484434	This study
12CN.HNCS501137	Hunan (Changsha)	2012	24	M	MSM	1.1-kb pol (pro-RT)	KJ484435	This study
11CN.LNSY300876	Liaoning (Shenyang)	2011	45	M	Heterosexual	NFLG	KJ484436	This study

M, Male; MSM, men-who-have-sex-with-men; NFLG, near full-length genome.

**Figure 2:**
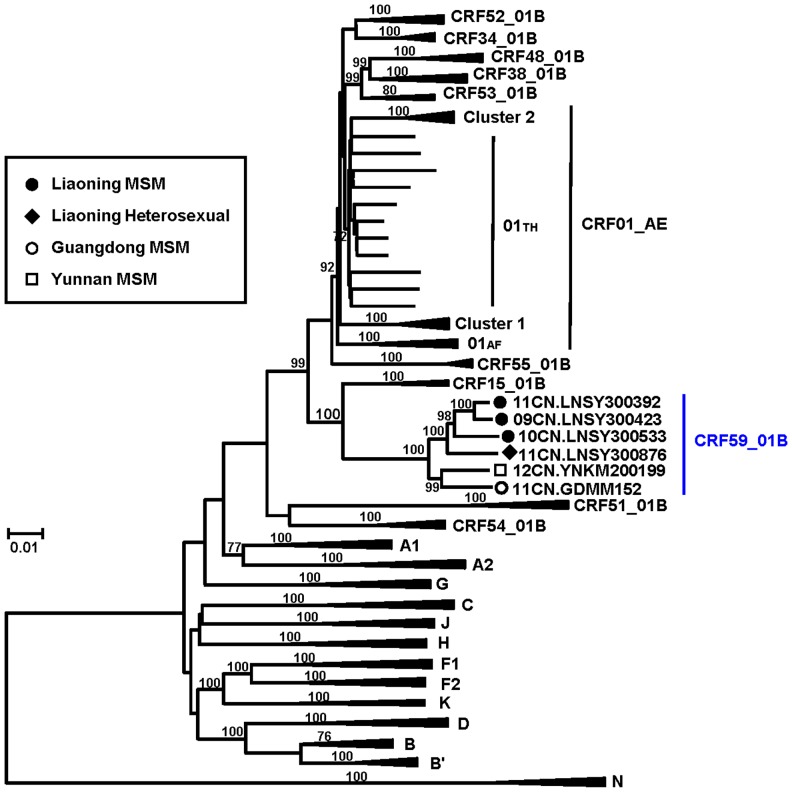
Neighbor-joining tree analysis of the near full-length nucleotide sequences of CRF59_01B. The neighbor-joining tree was constructed using the near full-length nucleotide sequences (8.8 kb) (HXB2: 790–9600 nt) of CRF59_01B strains identified in six epidemiologically-unlinked individuals [five MSM (Bi) and one heterosexual] from China (Table 1). These strains are compared with the reference sequences of all known subtypes/sub-subtypes as well as CRFs relevant to this study, including CRF15_01B, CRF34_01B and CRF52_01B from Thailand; CRF51_01B from Singapore; CRF33_01B, CRF48_01B, CRF53_01B and CRF54_01B from Malaysia; and CRF55_01B from China (http://www.hiv.lanl.gov/content/index). Bootstrap values (>70) of the respective nodes are indicated. 01th = Thailand CRF01_AE; 01af = African CRF01_AE; Chinese MSM clusters 1 and 2 = CRF01_AE variants associated with transmission among MSM in China.

**Figure 3:**
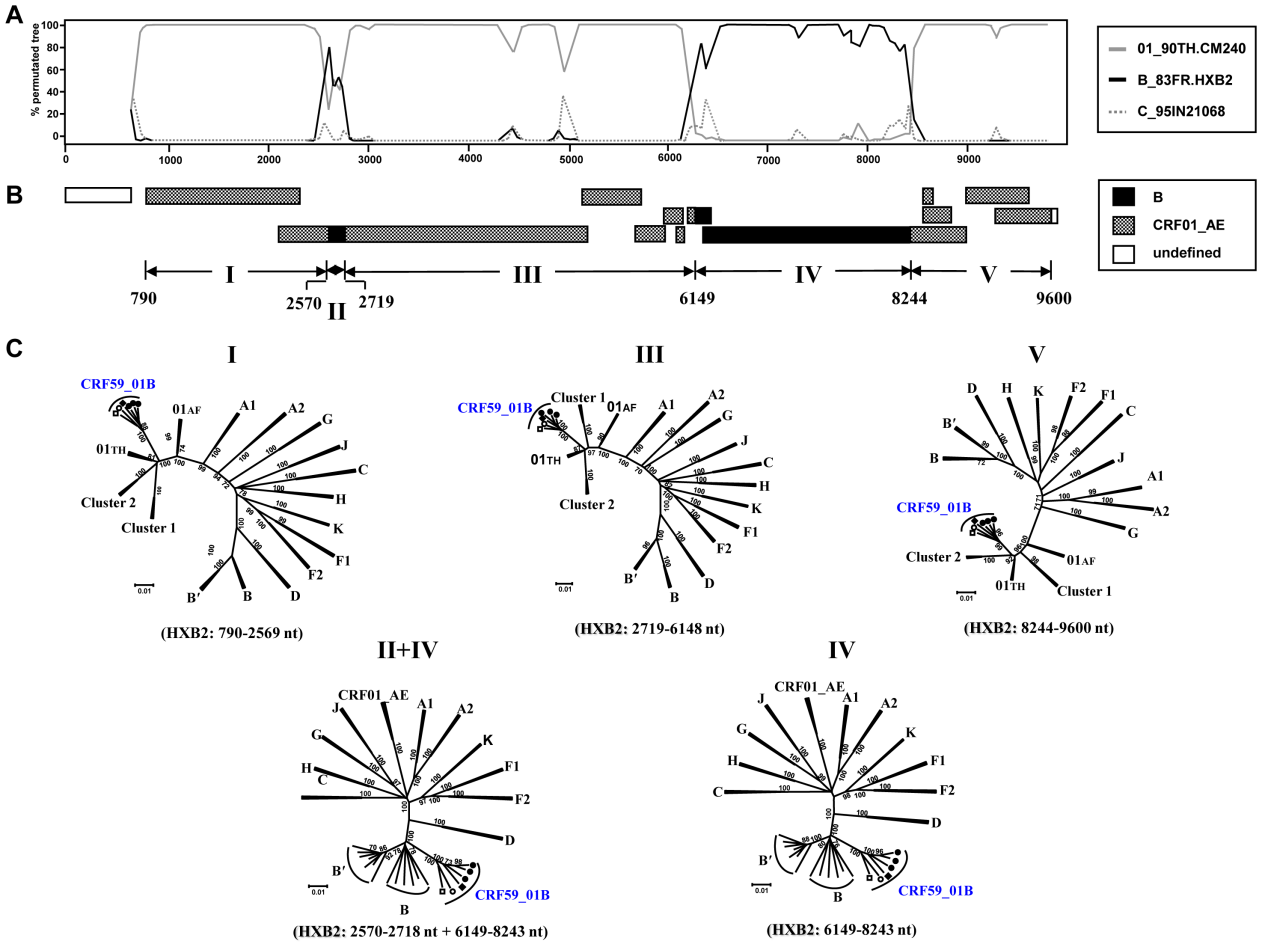
Recombination breakpoint analyses of CRF59_01B. (A) Bootscanning plot analysis. Analyses were performed using CRF01_AE (90TH.CM240) and subtype B (83FR.HXB2) as parental subtypes, and subtype C (95IN21068) as an out-group with a moving window of 350 nt with a step of 50 nt. Numbers represent nucleotide positions relative to the HXB2 genome. (B) The deduced subtype structure. Black = subtype B (of U.S.-European origin); gray = CRF01_AE; blank = no sequence data available. (C) Subgenomic phylogenies estimated using the neighbor-joining method from alignments representing Regions I, III, V (CRF01_AE) and the concatenated II+IV (subtype B) region. Bootstrap scores greater than 70% are indicated at corresponding nodes. 01th = Thailand CRF01_AE; 01af = African CRF01_AE; clusters 1 and 2 = CRF01_AE variants associated with transmission among MSM in China.

To define the detailed subtype structure of these strains, we determined the NFLG sequences from the plasma RNA samples. We successfully amplified and determined the NFLG sequences of six of the seven study subjects (all except 12CN.HNCS501137 from Hunan) (Table 1). As shown in Figure 2, a total of six NFLG sequences (five from MSM; one from a heterosexual male) (Table 1) formed a distinct monophyletic cluster that is distinct from any other known subtype or CRF. Recombination breakpoint analysis revealed that these six NFLG sequences shared identical recombinant structures in which two subtype B regions (nucleotide position 2570–2718 in the *pol* region and 6149–8243 nt in the region relative to the HXB2 genome) were located in a CRF01_AE backbone (Figure 3). The recombinant structure is distinct from any other known CRFs comprising CRF01_AE and subtype B, including CRF15_01B [22], CRF33_01B [23], CRF34_01B [24], CRF48_01B [25], CRF51_01B [3], CRF52_01B [4], CRF53_01B [5], CRF54_01B [6], and CRF55 01B [7]. Subregion tree analyses further confirmed the parental origins of each region of the recombinant genome as follows (Figure 3C): Region I (HXB2: 790–2569) = CRF01_AE; Region II (HXB2: 2570–2718) = B; Region III (HXB2: 2719–6148) = CRF01_AE; Region IV (HXB2: 6149–8243) = B; Region V (HXB2: 8244–9600) = CRF01_AE. Subregion tree analyses also indicated that the subtype B regions (Region II+IV) were of U.S.-European origin and not the type B′ (Thai variant of subtype B) [26], [27] lineage associated with blood-borne epidemics in Asia [28]. Additionally, the CRF01_AE regions were found in the Thailand CRF01_AE radiation and were not related to the CRF01_AE variants (clusters 1 and 2) that we recently identified among MSM in China [29]. We designated these novel CRF01_AE/B recombinants as CRF59_01B [30].

### Evolutionary Characteristics of CRF59_01B

**Figure 4:**
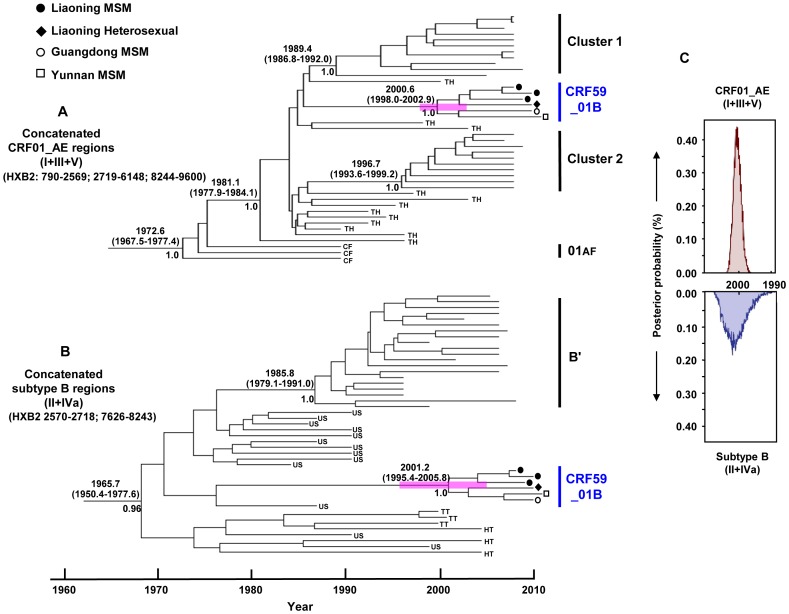
Maximum clade credibility (MCC) trees of CRF59_01B. The MCC tree was obtained by Bayesian Markov Chain Monte Carlo (MCMC) analysis of the concatenated CRF01_AE [(Regions I+III+V) (HXB2: 790–2569; 2719–6418; 8244–9600)] regions and subtype B [(Regions II+IVa) (HXB2: 2570–2718; 7626–8243)] regions, using a relaxed clock model in GTR+G4 with a constant coalescent model. Analyses were implemented in BEAST v.1.6.0. HIV-1 subtype C sequences are used as an out-group. The medians of the estimated tMRCAs with 95% highest probability density (HPD) (in parentheses) and the posterior probability (>0.95) of the nodes relevant to this study are indicated. (C) The distribution of the posterior probability of the estimated tMRCAs for CRF59_01B as well as the related CRF01_AE lineages (top) and subtype B lineages (bottom).

To estimate the timeline of emergence of CRF59_01B, we performed Bayesian molecular clock analyses on the CRF01_AE regions [Regions I (HXB2: 790–2569 nt), II (HXB2: 2719–6418 nt), III (HXB2: 8244–9600 nt) and concatenated genome regions for CRF01_AE (Regions I+III+V)(HXB2: 790–2569; 2719–6418; 8244–9600)] and on the subtype B regions [Region IVa (HXB: 7626–8243 nt) and the concatenated subtype B region (Regions II+IVa) (HXB2: 2570–2718; 7626–8243)] (Figure 4), respectively. Analyses were performed by using a relaxed molecular clock approach. Since the most recent common ancestor estimates (tMRCAs) using individual or combined regions for CRF01_AE [Regions I, III, V or the concatenated CRF01_AE region (I+III+V)] and subtype B regions [Regions IVa or the concatenated subtype B region (II+IVa)] yielded essentially similar results. For simplicity, we are showing the MCC trees for only the concatenated CRF01_AE region (I+III+V) and the subtype B (II+IVa) region (Figure 4).

As shown in Figure 4, the estimated tMRCAs for concatenated CRF01_AE regions (Regions I+III+V) and subtype B regions (Regions II+IVa) were 2000.6 [95% highest probability density (HPD): 1998.0, 2002.9] and 2001.2 [95% highest probability density (HPD): 1995.4, 2005.8], respectively (Figure 4). These resulting estimated tMRCAs for the CRF01_AE and subtype B regions are consistent with each other (Figure 4C). This suggests that the recombination generating CRF59_01B from parental lineages of subtype B and CRF01_AE occurred around the year 2001. In contrast, the estimated tMRCAs for Chinese MSM CRF01_AE cluster 1 [1989.4 (1986.8–1992.0)] and cluster 2 [1996.7(1993.6–1999.2)] are significantly earlier than those of CRF59_01B (Figure 4).

## Discussion

The large-scale national survey we conducted on HIV-1 strains circulating among MSM in China (Figure 1) identified a new CRF that we designated CRF59_01B (Figure 2, 3). CRF59_01B is the second CRF (after CRF55_01B [7]) to circulate primarily among MSM in China. This is the third CRF (after CRF51_01B from Singapore [3] and CRF55_01B [7]) to be identified among MSM in Asia. The appearance of this CRF reflects the recent upsurge of disease activity among MSM throughout the Chinese regions we studied [31]. As shown in the subregion trees (Figure 3C), the subtype B regions of CRF59_01B are of U.S.-Eurpoean origin, not of the subtype B′ (Thai variant of subtype B), which is associated with blood-borne epidemics in Asia. Additionally, CRF01_AE regions were found in the Thai CRF01_AE radiation and are not related to any CRF01_AE variants (clusters 1 and 2) that were recently identified among MSM in China [17]. As demonstrated in previous studies [13], subtype B was the predominant strain among MSM in China initially, but CRF01_AE has showed increasing prevalence among Chinese MSM in recent years. The co-circulation of these two HIV-1 lineages has led to the generation of various recombinants between the Thai CRF01_AE and subtype B strains, including CRF55_01B and CRF59_01B. These recombinants are distinct from other known CRFs consisting of CRF01_AE and subtype B′, including CRF15_01B and CRF34_01B from Thailand; CRF33_01B, CRF48_01B, CRF53_01B, CRF54_01B from Malaysia; and CRF52_01B from Thailand and Malaysia.

While CRF55_01B circulated widely among MSM in southern Chinese provinces, accounting for more than 10% of HIV-1 infections among MSM (X. Han et al., un- published data), CRF59_01B showed limited circulation among Chinese MSM. Although only a few CRF59_01B strains have been identified to date, they have been detected among MSM in various regions in China: 1.1% (3 of 263) in Liaoning from 2008 to 2012; 1.5% (1 of 68) in Hunan from 2010 to 2012; 2.5% (1 of 40) in Guangdong from 2011 to 2012; 1.5% (1 of 67) in Yunnan in 2012 (Figure 1B). These detections may suggest that an unknown focus of CRF59_01B is present outside the study sites. Alternatively, CRF59_01B has a very limited circulation, but the high mobility exhibited by the MSM population may explain the strain's sporadic and diffuse distribution across China.

According to the behavior studies [32], [33], most MSM lack a basic knowledge about HIV/AIDS and usually have multiple sexual partners with whom they exhibit unprotected sexual behavior. Indeed, the rate of infection of sexually transmitted diseases, such as syphilis, is remarkably high in many cities: 27.7% in Nanjing and Yangzhou [34], 31.1% in Shenyang [35], and 14.3% in Harbin [36]. These factors make it possible for MSM to experience multiple HIV infections and superinfections, facilitating the emergence of new recombinant strains and their rapid dissemination across China. The emergence of CRF55_01B and CRF59_01B suggests that new recombinant forms comprising the CRF01_AE and subtype B lineages are actively being generated among MSM in various regions of China.
